# AI-driven early infectious disease detection in Dutch primary care using BERT and ERNIE

**DOI:** 10.1038/s41746-025-02278-7

**Published:** 2025-12-23

**Authors:** Maarten Homburg, Gijs Danoe, Marjolein Y. Berger, Tim olde Hartman, Jean Muris, Andreas Voss, Axel Hamprecht, Maarten F. Brilman, Lilian L. Peters, Matthijs S. Berends

**Affiliations:** 1 Department of Primary and Long-term Care, University of Groningen, University Medical Center Groningen, Groningen, The Netherlands; 2 Department of Medical Microbiology and Infection Prevention, University of Groningen, University Medical Center Groningen, Groningen, The Netherlands; 3 Institute of Medical Microbiology and Virology, Carl von Ossietzky Universität, Oldenburg, Germany; 4 Department of Primary and Community Care, Radboud Institute for Medical Innovation, Radboud University Nijmegen Medical Center, Nijmegen, The Netherlands; 5 Department of General Practice, Maastricht University, Maastricht, The Netherlands; 6 Amsterdam UMC Location Vrije Universiteit Amsterdam, Midwifery Science, Amsterdam, The Netherlands; 7 Midwifery Academy Amsterdam Groningen, Inholland, Amsterdam, The Netherlands; 8 Department of Medical Epidemiology, Certe Foundation, Groningen, The Netherlands

**Keywords:** Infectious diseases, Computational models, Public health

## Abstract

Early detection of emerging infectious diseases is essential for timely public health interventions, yet traditional surveillance systems often miss early, non-specific signals. This study introduces Early Recognition using Neural Information Encoding (ERNIE), an autonomous, disease-agnostic natural language processing framework that applies unsupervised language modelling to unstructured primary care data to detect atypical disease clusters without predefined diagnostic labels. Trained on northern Netherlands data and validated using external datasets, ERNIE identified early COVID-19-like clusters before the first confirmed case, detected RSV-indicative patterns during the 2021 surge, and flagged simulated West Nile Virus cases, while remaining stable during controls (consultation-level recall 0.97; cluster precision 0.82; cluster recall 0.90). By analysing routine clinical text, ERNIE enables early and interpretable detection of emerging health threats without reliance on diagnostic codes or laboratory confirmation. Beyond this, the framework holds potential for identifying atypical clinical patterns in other domains, supporting scalable, AI-driven monitoring across health systems.

## Introduction

Emerging infectious diseases pose a continuous threat to global health systems, underscoring the need for optimised, proactive surveillance strategies that enable early detection and targeted response^1^. Existing systems, such as the Electronic Surveillance System for the Early Notification of Community-Based Epidemics (ESSENCE) from the US Centers for Disease Control and Prevention (CDC) and the European Surveillance Portal for Infectious Diseases (EpiPulse) operated by the European Centre for Disease Prevention and Control (ECDC), mainly rely on structured data sources such as emergency department chief complaints, diagnostic codes, pharmacy sales, and school absenteeism records^2–5^. Although these platforms support syndromic and event-based surveillance at national and international levels, they typically do not incorporate the rich, longitudinal patient-level information available in routine general practice, particularly early and undifferentiated clinical presentations that fall outside predefined diagnostic categories. This limits their ability to detect emerging outbreaks presenting with vague or non-specific symptoms in early stages. Consequently, such detection gaps could result in substantial clinical and economic burdens. For example, undetected legionellosis cases in New Zealand contributed to an estimated annual hospitalisation cost of over NZ$2.1 million, with a median inpatient stay of 6 days and intensive care costs exceeding NZ$57,000 per case. The study emphasised that this preventable disease imposes a substantial burden on the healthcare system, particularly among older adults, and that improvements in (early and autonomous) surveillance could severely reduce hospitalisation rates and associated expenditures^6^.

To overcome such limitations, surveillance systems should capture emerging infections as early as possible in the patient’s care-seeking trajectory. In the Netherlands, general practitioners (GPs) serve as gatekeepers of the healthcare system, functioning as the first point of contact for both acute and non-acute health concerns^7^. Unlike hospitals, where patients typically present with more severe disease, primary care records contain early-stage symptoms and mild presentations, which are key factors for detecting emerging outbreaks before they escalate. Dutch GPs typically document encounters using structured International Classification of Primary Care (ICPC) codes alongside detailed narrative entries in free-text fields, creating a rich source of clinical data^8^. Though recorded within predefined electronic health record (EHR) sections, this content remains unstructured from a data science perspective, lacking standardised formats or controlled vocabularies. While the structure and extent of free-text documentation vary internationally, the Dutch system offers an especially dense and consistent textual record, making it well-suited for text-based surveillance research.

However, the unstructured nature of GP text data presents significant analytical challenges. In the Netherlands, primary care surveillance is currently based on weekly counts of predefined syndromes and health problems coded using the ICPC across a large network of general practices^9^. Internationally, similar systems rely on structured classifications such as ICPC or the International Classification of Diseases (ICD)^10,11^. These approaches depend on structured data inputs and predefined syndromes, limiting their suitability for detecting novel or atypical presentations. For newly emerging diseases, a specific code may not yet exist, or no registration guidelines may be available. Although primary care-based surveillance is well established, it remains largely code-based and does not leverage the diagnostic richness embedded in unstructured consultation text. Advanced AI-driven methods, particularly natural language processing (NLP), offer a way to extract clinically meaningful insights from such data in a disease-agnostic manner.

Recent advances in NLP, particularly Bidirectional Encoder Representations from Transformers (BERT), have transformed the ability to process and interpret unstructured text^12^. While BERT has been widely used in supervised learning tasks, where models are trained on predefined diagnostic labels to classify or extract information from text, it also enables unsupervised and self-supervised approaches^13–16^. A promising next step is to leverage BERT’s semantic representations in an unsupervised framework to detect emerging disease patterns independently of structured labels. Despite the availability of powerful AI techniques and rich primary care datasets, literature has shown no existing method able to autonomously detect and characterise clusters of emerging infectious diseases in primary care settings. Addressing this gap, we introduce Early Recognition using Neural Information Encoding (ERNIE), a next-generation AI-driven surveillance framework that moves beyond existing NLP-based methods. The ERNIE framework integrates a multi-step modelling pipeline, combining autoencoders for anomaly detection, clustering algorithms to group detected anomalies, key term extraction for interpretability, and cluster visualisation to enhance insights. This study aims to develop, evaluate, and externally validate ERNIE as a generalisable surveillance framework for the early and autonomous detection and characterisation of emerging infectious disease outbreaks in primary care.

## Results

### Data source and population

After data cleaning and applying the inclusion criteria, 446,354 unique consultations from the Academic GP Development Network (Academisch Huisartsen Ontwikkel Netwerk, AHON) database, covering the period from January 1, 2015, to December 31, 2023, were retained for analysis. For external validation, the same criteria were applied to two independent datasets, yielding 109,856 consultations from the eastern region of the Netherlands and 367,960 consultations from the southern region. Additional consultation characteristics are detailed in Supplementary Table 1.

### Testing and validation of the ERNIE framework

**Fig. 1:**
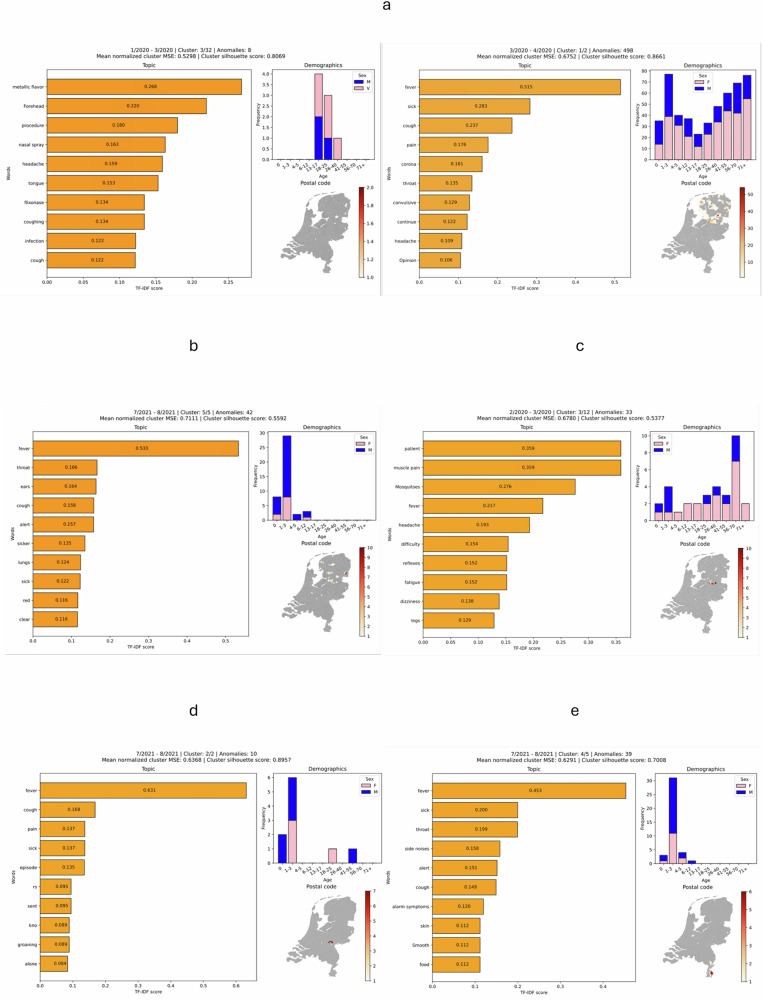
Cluster visualisations of detected anomalous patterns. **a** Around the onset of the COVID-19 pandemic (AHON, February–March 2020). **b** During the RSV surge in the summer of 2021 (AHON, July 2021). **c** Following the introduction of a simulated West Nile Virus cluster. **d** External validation during the RSV surge in the summer of 2021 (RUMC, July 2021). **e** External validation during the RSV surge in the summer of 2021 (MUMC + , July 2021).

The performance of the ERNIE framework was tested and validated through multiple scenarios, encompassing real-world primary care data, simulated disease outbreaks, and independent external datasets. Detailed visualisations of identified clusters for each validation scenario are presented in Fig. 1 (panels a–e). Each panel displays a representative cluster, including its topic composition, demographic features, geographic distribution, and clustering metrics. In addition, the number of detected clusters for the respective period and the number of anomalies assigned to the depicted cluster are indicated in the panel headers, providing context on overall model activity during each scenario. The full set of identified clusters is displayed in Supplementary Table 2.

#### Real World Data

The framework was applied to AHON data from January and February 2020, preceding the first confirmed COVID-19 case in the Netherlands on February 27. It identified 32 clusters. One of these clusters consisted of eight anomalous consultations (mean MSE: 0.53, silhouette score: 0.81) with keywords including metallic flavour, cough, headache, and infection. The other clusters varied in size, keyword profile, and demography.

During March 2020, directly following the initial phase of the epidemic, two clusters were found; a large cluster of 498 anomalous consultations (mean MSE: 0.68, silhouette score: 0.87), characterised by keywords including fever, cough, headache, throat, and corona. This cluster was observed across all demographic groups and exhibited a broader geographic distribution (Fig. 1a). The second cluster (nine anomalous consultations) consisted of similar symptoms.

During July 2021, the framework detected five distinct clusters. Three of these (mean MSE’s: 0.71, silhouette scores: 0.56–0.85), primarily affected young children aged zero to three years, with keywords including fever, cough, throat, lungs, and ears (Fig. 1b). This period coincided with a documented surge in RSV infections in the Netherlands.

#### Simulation of disease outbreak

The framework analysed 30 simulated consultations from February 2020 presenting West Nile virus (WNV)-like symptoms. Despite elevated background activity preceding widespread COVID-19 confirmation, ERNIE identified a distinct cluster of 33 anomalous consultations (mean MSE: 0.69; silhouette score: 0.54) characterised by keywords such as mosquitoes, fever, muscle pain, dizziness, and fatigue (Fig. 1c). These features correspond closely to the simulated symptom profiles.

The anomaly detection stage correctly identified 29 of the 30 simulated consultations (recall 0.97). Of these, 27 were grouped into this same cluster containing 33 consultations in total (cluster precision 0.82; cluster recall 0.90).

#### Outbreak-free reference period

In the July 2022 dataset, corresponding to a period with no documented infectious disease outbreaks, the model was applied to assess specificity. No distinct anomalous clusters were detected.

#### External validation

The framework was independently applied to two external datasets to evaluate its performance across regions, focusing on the RSV surge in July 2021.

Radboud University Medical Center (RUMC) dataset (eastern Netherlands): the framework detected two respiratory clusters (mean MSE’s: 0.64–0.66, silhouette scores: 0.72–0.90), both primarily affecting young children aged zero to three years, with keywords including fever, sick, cough, breathing, and RS (Fig. 1d).

Maastricht University Medical Center (MUMC + ) dataset (southern Netherlands): five clusters were identified, of which three respiratory clusters predominantly affecting young children (mean MSE’s: 0.63–0.64, silhouette scores: 0.65–0.77), with keywords such as fever, cough, and throat (Fig. 1e).

## Discussion

This study presents the development, testing, and validation of a novel, unsupervised surveillance framework, ERNIE, that detects and characterises atypical consultation patterns from routine primary care data, enabling the identification of both naturally occurring and simulated signals without reliance on predefined diagnostic codes. By applying NLP techniques to unstructured primary care data, the ERNIE framework autonomously identified anomalous clusters, suggesting its potential to detect deviations in consultation patterns that may precede formal outbreak recognition. The framework was validated on independent datasets across geographically distinct regions, indicating generalisability and the potential for scalable, real-time syndromic surveillance in clinical practice.

Consistent with ECDC/CDC guidance for event-based surveillance, ERNIE is evaluated as a signal detection system rather than a case classifier. Surveillance utility was assessed using operational criteria recommended for early-warning systems: timeliness, predictive value of alerts, simulation/spike-in detectability, alert burden/false-alarm workload, replication on independent data^17,18^.

The ERNIE framework identified deviations aligned with various known outbreaks. First, regarding timeliness, prior to the first confirmed COVID-19 case in the Netherlands, the framework identified a small but cohesive cluster in February 2020 (MSE: 0.53; silhouette: 0.81), with symptoms later linked to COVID-19. Most notably, “metallic flavour”, which had the highest TF-IDF score within the cluster, was among the characteristic symptoms retrospectively recognised as indicative of COVID-19 infection^19^. This example, solely derived from free-text entries, illustrates the added value of text-based surveillance in capturing early, uncoded clinical signals. Across the pre-confirmation window, ERNIE identified 31 additional anomalous clusters, largely small and partially overlapping. This dispersion is consistent with an early, uncoded phase and may have been amplified by shifts in healthcare-seeking or documentation amid rising pandemic concern. These findings align with prior hypotheses suggesting that patients with COVID-19 symptoms had already presented to primary care providers before the first confirmed hospitalisations^20,21^. This further underscores the value of primary care surveillance for detecting outbreaks earlier than hospital-based systems, as symptom onset often preceded hospital admission by 1–2 weeks^22^. By March 2020, these early signals had consolidated into a much larger and more coherent cluster (MSE: 0.68; silhouette: 0.87), most likely corresponding with widespread viral transmission^23^. This progression illustrates a central trade-off in outbreak detection: early signals offer greater timeliness but less internal cohesion, whereas later clusters are more stable and interpretable but emerge after substantial transmission has occurred. Surveillance systems must balance these opposing priorities.

Second, regarding predictive value of alerts, ERNIE was applied to consultations from July 2021, coincident with the nationally documented summer RSV surge (RIVM), which was atypical relative to the usual autumn–winter season^24^. The framework identified five anomalous clusters, three of which consisted of young children (0–3 years) with key terms such as fever, cough, and throat, lung, and ear complaints, which are clinical features commonly associated with RSV^25^. While interpretation should remain cautious, the timing, age distribution, and symptom profiles of these paediatric clusters strongly suggest RSV circulation. These findings further support ERNIE’s operational capacity to flag smaller outbreaks when they emerge in primary-care texts. Two smaller, non-specific older-adult clusters were triaged as background.

Third, regarding simulation/spike-in detectability, simulation of a West Nile Virus (WNV) outbreak demonstrated the framework’s ability to detect symptomatic clusters of previously unseen conditions. The model successfully identified a distinct cluster corresponding to the 30 manually introduced consultations, with key terms such as mosquitoes, fever, muscle pain, dizziness, and fatigue. Quantitatively, the anomaly detection stage correctly identified 29 of the 30 simulated consultations (recall 0.97), of which 27 were grouped into a single coherent cluster of 33 consultations in total (cluster precision 0.82; cluster recall 0.90). These results confirm that ERNIE reliably detects and groups novel clinical presentations without prior examples, providing measurable evidence of signal discrimination. The ability to recover this cluster with high recall and precision under high epidemic pressure (i.e., during early COVID-19 activity) further supports the framework’s robustness and suitability for integration into routine surveillance workflows.

Fourth, regarding alert burden/false-alarm workload, the framework was applied to consultation data from July 2022, a period with no reported unusual infectious disease activity. No anomalous clusters were detected, suggesting a low risk of false positive signals under stable epidemiological conditions. However, the absence of labelled ground truth limits definitive performance quantification. Importantly, detection of a deviation during a period without a reported outbreak does not necessarily indicate model failure, as it may reflect unrecognised or subclinical patterns in primary care. Future implementation studies should investigate threshold calibration and the definition of meaningful alerts in operational contexts, where retrospective validation remains inherently constrained.

Fifth, regarding replication on independent data, external validation of the ERNIE framework was performed using independent datasets from primary care research networks in the eastern and southern regions of the Netherlands. Despite being trained exclusively on data from the northern region, the framework effectively identified anomalous respiratory clusters in both external datasets during the 2021 RSV surge. While the detected clusters were consistent with known RSV symptomatology and demographics, the key finding is that the framework successfully generalised to geographically distinct primary care populations.

Taken together, these results support the ERNIE framework as an early-warning surveillance tool aligned with ECDC/CDC principles for event detection. The framework prioritises unsupervised, pattern-level discovery, interpretability, timeliness, stability in quiet periods, and robustness across settings. Although ERNIE was not trained to detect specific diseases (e.g., COVID-19, RSV, WNV), it successfully identified deviations in consultation patterns based on anomalies in free-text notes, seasonal trends, and demographic patterns.

Beyond the content of individual clusters, the overall volume of detected anomalies and clusters during a given period can itself serve as an indicator of epidemiological change. In the current study, cluster examples were selected based on retrospective knowledge of known outbreaks, but in practice, the framework is designed to surface unexpected patterns without prior assumptions. Periods of increased clustering activity may point to emerging syndromes or shifts in healthcare-seeking behaviour, while individual clusters provide interpretable insights into their potential drivers. This distinction underscores ERNIE’s dual utility as both an early warning system and a tool for contextual exploration.

Building on its retrospective validation, ERNIE’s architecture is well suited for integration into clinical and public health workflows, enabling timely and scalable outbreak detection. In primary care, the detection of unexpected symptom clusters could, for example, prompt targeted diagnostic workups or heightened patient monitoring. Regionally, geographically concentrated clusters may inform hospital preparedness or triage planning. At the national level, ERNIE could complement existing structured surveillance systems by offering autonomous, text-based anomaly detection. Such capacity could provide lead time for mitigation or containment, particularly during the early stages of outbreaks, potentially enabling earlier diagnostic confirmation, targeted interventions, or patient isolation, and thereby reducing transmission and alleviating pressure on healthcare services. As a proof of concept, ERNIE demonstrates the methodological feasibility of identifying early, undifferentiated clinical signals in routine care data. Future implementation research will be needed to evaluate its real-time integration, communication pathways, and governance frameworks in practice.

Model outputs were designed to support interpretation with minimal technical expertise. Each cluster includes the number of anomalies, average MSE, silhouette score, age and sex distribution, postal code-level geography, timeframe, and salient keywords extracted from clinical text. These visual and numerical summaries enable rapid assessment of clinical plausibility and public health relevance.

These features position ERNIE as a flexible alternative to conventional surveillance approaches, which face several well-documented limitations. While traditional surveillance systems rely on diagnostic codes or structured symptom counts, they are often limited by reporting delays, rigid classification schemes, and retrospective aggregation, which may fail to capture subtle shifts in clinical presentation^26^. Syndromic systems can offer earlier signals but frequently lack clinical specificity and are prone to oversensitivity when based on non-clinical inputs^27^. Recent NLP-based approaches have shown promise in analysing unstructured text but generally rely on supervised learning tailored to predefined conditions, limiting flexibility in the face of novel or evolving syndromes^28^. Together, these capabilities strengthen the case for integrating free-text analysis into routine surveillance practice.

Several limitations should be acknowledged. The framework’s performance is inherently linked to the quality and consistency of GP documentation, which can vary in terminology, completeness, and structure. Some records contain rich clinical narratives, while others rely on shorthand or omit relevant symptoms, potentially affecting anomaly detection. These limitations underscore the importance of comprehensive and standardised documentation practices to optimise model output.

Furthermore, as an unsupervised system, ERNIE identifies statistical deviations without establishing causality. Clinical and epidemiological expertise remains essential to contextualise the findings and assess their significance. This interpretive requirement is inherent to surveillance solely based on unsupervised detection models, thus highlighting the need for multidisciplinary collaboration to ensure responsible application.

The study lays the foundation for broader implementation and future research. Scalability and generalisability are essential for deploying ERNIE across clinical and public health settings. The framework generalised well to external datasets within the Netherlands, supporting broader national deployment. International implementation is also plausible, provided access to language-specific or multilingual BERT models and regionally representative data for retraining. Nationally, ERNIE may further benefit from a Dutch, domain-specific (i.e., GP) language model trained on clinical texts. Although such a resource was not available at the time of this study, its development would meaningfully support future iterations of the framework. More broadly, this underscores the importance of investing in medical language models that capture the linguistic and contextual nuances of clinical text, which can enhance the performance and adaptability of frameworks like ERNIE across diverse healthcare settings. With these technical foundations in place, attention can turn to questions of operational deployment. Initial implementation is particularly feasible within academic or sentinel primary care networks, where data governance structures and privacy safeguards are already established. These environments offer a practical context for early calibration and feedback, enabling iterative refinement of ERNIE’s configurations and interpretive protocols. Real-world evaluation in such settings could inform broader national or international deployment.

As with all unsupervised tools, ERNIE flags deviations but does not prescribe action. Thresholds can be tuned to favour higher specificity or broader sensitivity, depending on user requirements. Implementation studies should explore how different configurations affect alert frequency and downstream utility. While ERNIE’s transparency promotes trust, successful use will require defined protocols for action, appropriate interpretive training, and public health frameworks to navigate the ethical complexities of acting, or choosing not to act, on uncertain early signals.

As labelled data become available, a supervised classification model could be trained to assign detected anomalies to known disease categories. Beyond infectious disease surveillance, ERNIE may also support detection of symptom patterns associated with other diseases, where earlier recognition could improve outcomes.

In summary, this study introduces a novel, scalable framework that applies unsupervised language modelling to unstructured primary care data for autonomous outbreak detection. ERNIE demonstrated the ability to identify early and coherent clusters of anomalies without predefined disease labels or reliance on structured data. Through explainable outputs, the framework provides interpretable insights into symptomatology and population characteristics.

Beyond infectious disease detection, the ERNIE framework demonstrates potential for identifying emerging clinical patterns that fall beyond predefined diagnostic categories. By enabling early recognition of atypical symptom clusters in routine healthcare data, this approach may support broader applications across a range of surveillance domains. Therefore, the framework represents a significant advancement in the use of unstructured clinical text, enabling rapid, scalable, and transparent autonomous disease detection.

## Methods

### Data source and population

This study was conducted using consultation data derived from primary care EHRs from general practices participating in the AHON, managed by the University Medical Center Groningen (UMCG)^29^. At the time of the study, the AHON registry database contained longitudinal data from 515,761 patients from 85 general practices. Available data included physician consultation notes, date of consultations, ICPC-1 codes, medical history, medication prescriptions, demographic information and postal code. The study period spanned January 1, 2015, to December 31, 2023. Raw data was cleaned as described before^30^.

In the Netherlands, GPs are required to assign at least one ICPC code to each free-text consultation entry^8^. To identify consultations relevant to infectious diseases, three of the authors (two GPs: MH and MYB, and a microbiologist: MSB) independently reviewed all 1257 ICPC-1 codes for their relevance to infectious diseases. All three reviewers assessed the full set of ICPC-1 codes independently, after which cases of uncertainty were discussed within the research team and classified as included or excluded based on expert consensus. This process resulted in the selection of 102 ICPC-1 codes relevant to endemic and pandemic infectious diseases, covering respiratory, gastrointestinal, and neurological conditions, as well as general infection-related symptoms (Supplementary Table 3). All consultations linked to at least one of the selected ICPC-1 codes were extracted.

GP consultations follow a standardised documentation structure known as SOEP, which consists of four free-text fields. The Subjective (S) section captures patient-reported symptoms and complaints, while the Objective (O) section records clinical observations and measurements. The Evaluation (E) section includes diagnoses or clinical impressions, typically linked to an ICPC code, and the Plan (P) section outlines treatment plans, prescriptions, or follow-up recommendations^8^. For this study, a GP consultation was defined as any patient interaction that included at least one of the selected ICPC-1 code and documented entries in at least two of the four SOEP sections.

### Framework development

**Fig. 2:**
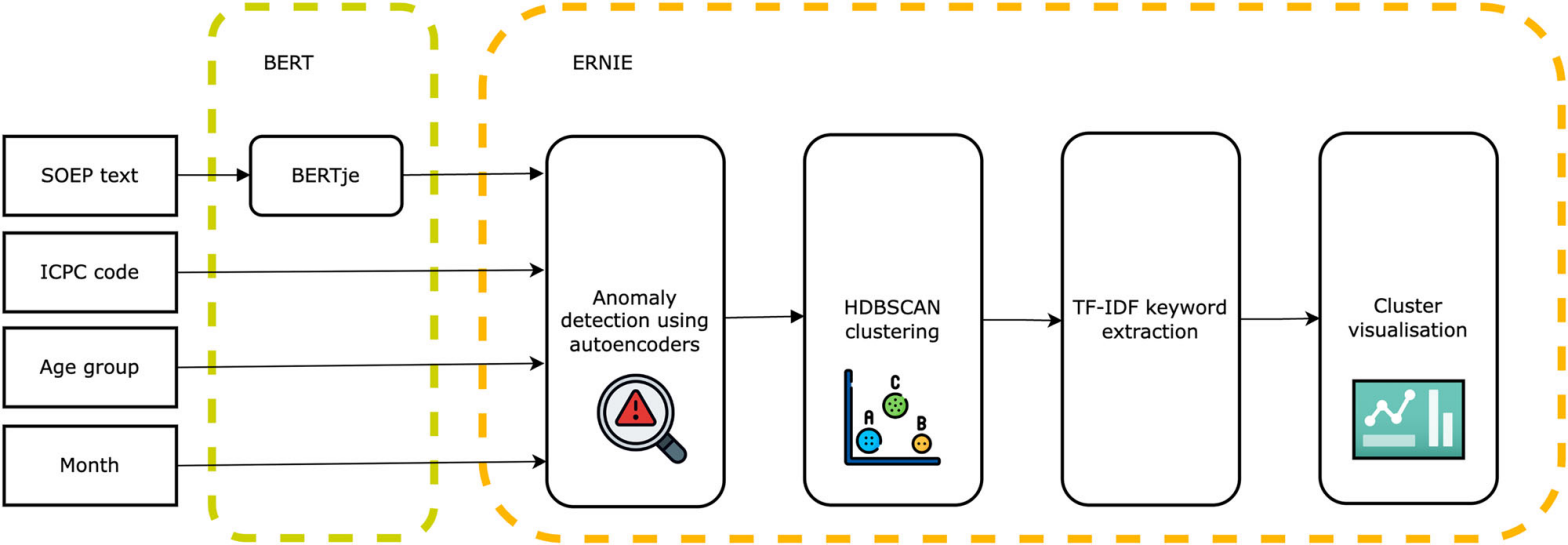
Schematic overview of the developed framework. The pipeline integrates SOEP texts, ICPC codes, age group, and consultation month. BERTje generates contextual embeddings from text, which are analysed using autoencoders for anomaly detection. HDBSCAN clusters anomalies, and TF-IDF extracts keywords for interpretability, with results visualised for analysis. Figure created by the authors using draw.io.

An NLP framework was developed for the early detection of atypical disease patterns in primary care data (Fig. 2). The framework builds upon BERT, a deep learning model pre-trained for context-aware text processing^12^. The ERNIE framework integrates (1) auto-encoders for anomaly detection, (2) Hierarchical Density-Based Spatial Clustering of Applications with Noise (HDBSCAN) for clustering, (3) Term Frequency - Inverse Document Frequency (TF-IDF) for cluster-level key term extraction and (4) cluster visualisation for enhanced insights.

#### Analytical objective and evaluation scope

ERNIE is an unsupervised, disease-agnostic signal detection framework that operates on routine GP free-text. Its outputs are atypical clusters of consultations (patterns) rather than case labels for specific diseases. Because ERNIE is not trained on disease-specific labels and no exhaustive ground-truth outcomes exist at the consultation level, typical case-level performance metrics such as sensitivity and specificity on real world data are neither identifiable nor meaningful in this setting. Accordingly, evaluation is conducted at the event (pattern) level, asking whether coherent, interpretable clusters arise during independently documented outbreak periods with useful lead time; whether the system remains quiescent in outbreak-free reference periods; whether clinically coherent spike-in simulations are surfaced as distinct clusters; and whether signals replicate across regions. These criteria reflect surveillance utility for early warning rather than diagnostic case ascertainment; further rationale is provided in the Discussion.

#### BERT-based text encoding for modelling

Consultation texts were converted into dense numerical representations, so-called embeddings, using BERTje, a Dutch-adapted version of BERT trained on extensive Dutch-language corpora^31^. This enabled capturing contextual relationships between words, which is essential for interpreting GP consultation notes. Non-textual features, including ICPC codes, age group, and month, were numerically encoded.

#### Anomaly detection

**Fig. 3:**
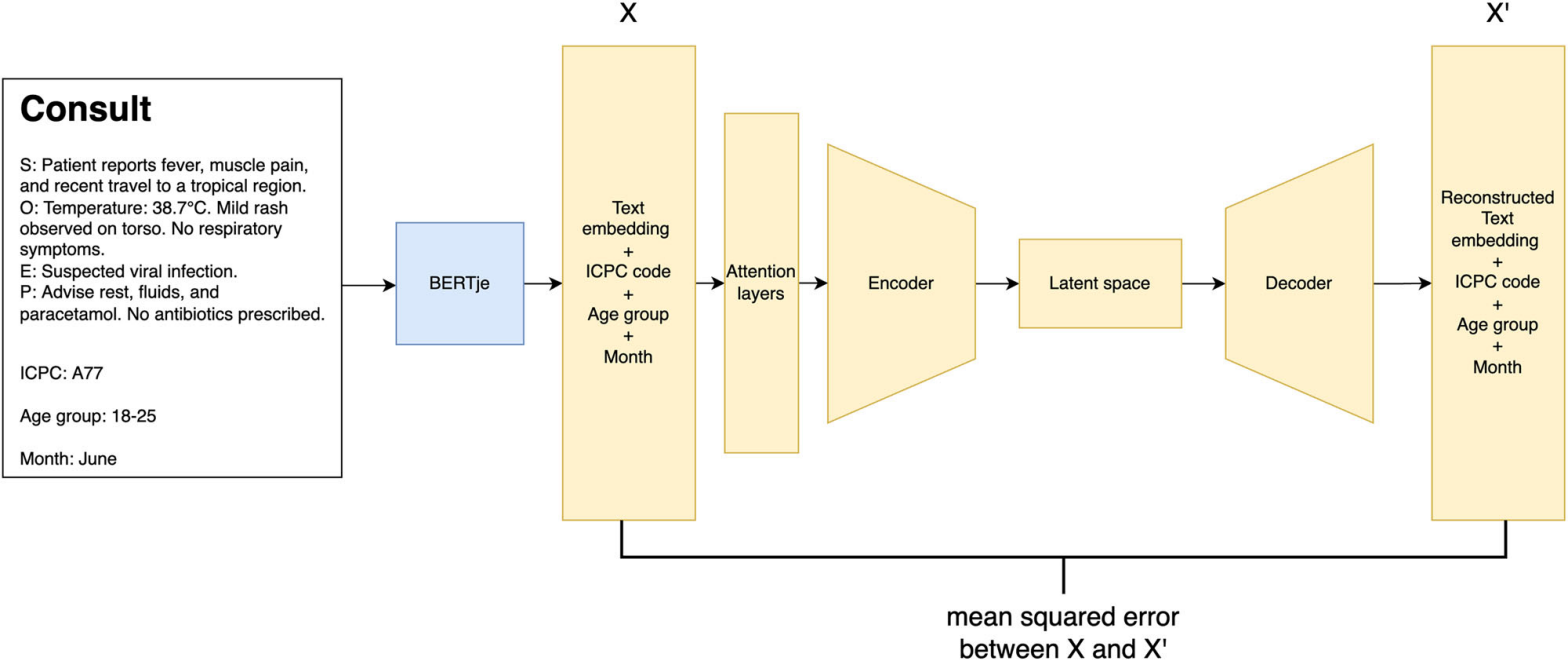
Schematic representation of a typical (fictive) consultation and the autoencoder framework. The figure illustrates how the autoencoder processes consultation data, learning to encode and decode information to identify deviations from typical consultation patterns. Consultations showing higher reconstruction errors are flagged as potential anomalies. Figure created by the authors using draw.io.

An autoencoder neural network was used for temporal anomaly detection in consultation data, schematically outlined in Fig. 3. The underlying idea of this approach is that, by reconstructing routine primary-care consultations, the model captures regularities in common symptom combinations and presentation patterns. Attention layers were applied before encoding to prioritise the most informative textual features. The autoencoder compresses consultation representations into a lower-dimensional latent space and reconstructs them from this internal representation. In theory, consultations that differ from such expected patterns, through uncommon symptom combinations or changes in textual structure, produce higher reconstruction errors and can therefore be flagged as potential anomalies. We applied a fixed threshold corresponding to the 90th percentile of the training reconstruction error distribution. This threshold is commonly used in autoencoder-based anomaly detection, as it allows for a broader yet still precise identification of anomalies^32^. Observations with reconstruction errors above this threshold were classified as anomalous.

#### Clustering of anomalies

Anomalous consultations identified by the autoencoder were clustered using HDBSCAN to uncover distinct patterns in clinical text that may signal emerging health concerns^33^. HDBSCAN defines cluster structures based on data density, where similar consultations form dense regions, of which outliers are classified as noise. Tuning parameters are detailed in Supplementary Table 4.

To enhance clustering efficiency, Uniform Manifold Approximation and Projection (UMAP) was applied to compress the BERTje representations for dimensionality reduction, preserving key text relationships while reducing computational complexity^34^. Anomalies were clustered based on these reduced representations, incorporating textual content and patient age while remaining independent of categorical variables such as ICPC codes. Clusters with at least five anomalies were considered meaningful based on expert consensus within the research group, while smaller clusters were classified as noise to ensure analytical robustness.

#### Key term extraction

After clustering, TF-IDF was applied to extract keywords that characterised each cluster^35^. Consultation texts within each cluster were aggregated, and TF-IDF identified terms that were both frequent within the cluster and distinctive compared to the overall dataset.

By assigning higher importance to words that occur frequently in a specific cluster but remain uncommon elsewhere, TF-IDF down-weights generic medical terms (e.g., *pain*, *fever*) while emphasising cluster-specific terms. This approach allowed defining clinical features, revealing potential symptom patterns, unexpected symptom combinations, or atypical presentations relevant to anomaly detection.

#### Cluster visualisation

The model output was visualised to summarise key cluster characteristics, including demographic distributions (e.g., age, gender, geographic location) and TF-IDF keyword summaries, highlighting dominant themes. To facilitate reporting in English, keywords were translated from Dutch using the OPUS-MT model^36^.

Clusters were evaluated using two interpretable key metrics, both ranging from 0 to 1. The normalised mean squared error (MSE) quantified the average reconstruction error, with higher values indicating greater deviation from routine patterns. The silhouette score assessed cluster cohesion, where higher values reflected stronger internal consistency among anomalies.

### Testing and validation of the ERNIE framework

The framework was trained on all consultations up to the start of the testing period, establishing a baseline of routine clinical patterns. Consultations from the testing period were excluded from training and used for evaluation. Model performance and statistical analyses were assessed using Python (version 3.8.12) and R (version 4.1.1; R Foundation for Statistical Computing). A detailed technical description of the framework, including hyperparameters and pre-processing steps, is provided in Supplementary Table 3.

Clusters illustrated in this manuscript were selected for interpretability and clinical relevance by clinical experts (MH/MSB); the complete, unfiltered clusters generated by the ERNIE framework are provided in Supplementary Table 2.

#### Real World Data

The framework’s generalisability and effectiveness were assessed using data from documented outbreaks in the same region as where the training data were gathered from. To assess its ability to detect emerging disease patterns, the framework was applied retrospectively to COVID-19 case peaks, both before the first confirmed cases in the Netherlands and during the initial outbreak^37^. Additionally, it was tested on the atypical summer surge of Respiratory Syncytial Virus (RSV) in 2021, when RSV, typically a winter disease, exhibited unseasonal activity^24^.

#### Simulation of disease outbreak

To validate the ERNIE framework’s ability to detect previously unobserved diseases, a simulated dataset was created using an infectious disease not known to have occurred in the Netherlands during the study period. To this end, West Nile Virus (WNV) was chosen as a test case for several reasons. First, it poses a credible risk for regional emergence. Second, it presents with non-specific early symptoms but can lead to severe complications (e.g., meningitis). Third, its distinct mosquito-borne transmission pattern introduces seasonal and ecological dynamics that may manifest as detectable anomalies.

An experienced general practitioner (MH) manually drafted 30 synthetic consultations in Dutch, using the standard SOEP structure as routinely used in all Dutch primary care records. These cases were informed by the World Health Organization (WHO) case definitions for West Nile virus (WNV)^38^, and included plausible symptom combinations, progression, and contextual details. The goal was not to simulate or recognise WNV specifically, but rather to test the model’s ability to detect and cluster a group of clinically coherent but non-coded, atypical presentations. To provide a stringent and operationally realistic challenge, the synthetic consultations were injected into the February 2020 data: a pre-confirmation window preceding widespread COVID-19 acknowledgment and characterised by elevated background activity, to evaluate detectability under high-noise conditions rather than in isolation. In this context, the specific pathogen was incidental: the key objective was to demonstrate that such outlier cases, even when expressed through free text and without explicit diagnostic labels, could be surfaced by the model as an emergent cluster.

To further assess performance, detection outcomes from this simulation were compared with the known injected cases, allowing calculation of precision and recall for both stages of the framework: (1) anomaly detection at the consultation level and (2) clustering of detected anomalies into coherent groups.

#### Outbreak-free reference period

To assess the risk of spurious cluster detection, the model was applied to a reference dataset from July 2022, a period during which no regional infectious disease outbreaks were reported based on available epidemiological data. This setup enabled evaluation of whether coherent clusters would be detected in the absence of confirmed outbreak signals, providing an indirect estimate of false positive risk.

#### External validation

After training and local testing, the ERNIE framework’s generalisability was externally validated using electronic health records from two independent academic research networks in the Netherlands. This evaluation on previously unseen datasets assessed the model’s robustness in detecting anomalies across different primary care populations and data sources, ensuring its applicability outside the training environment. Data were obtained from the Family Medicine Network, managed by RUMC, which covers the eastern region of the Netherlands, and the Research Network Family Medicine, managed by MUMC + , which covers the southern region^39,40^. The ERNIE framework was applied to these datasets to assess its ability to detect potential indicators of the atypical RSV surge in summer 2021 that occurred throughout the Netherlands.

#### Ethical considerations

This study complies with the Declaration of Helsinki. Data were obtained in a pseudonymised format, with no access to personally identifiable information. The Medical Ethics Review Committee of the University Medical Center Groningen reviewed the study protocol and confirmed that formal ethical approval was not required under Dutch law (reference: METc 2021/549).

## Supplementary information

Supplementary Information

## Data Availability

Access to the training data is subject to approval by the steering committees of the participating general practitioner networks. The dataset contains confidential patient health information and cannot be made publicly available due to privacy regulations. Formal approval is required, and additional conditions may apply.

## Code availability

The ERNIE framework is publicly available at https://github.com/gijsdanoe/berternie and released under the GNU General Public License v2.0 (GPL-2.0).
