# Supplementary material for: AI-driven early infectious disease detection in Dutch primary care using BERT and ERNIE
Source: NPJ Digit Med. 2025 Dec 23;9:92. doi: 10.1038/s41746-025-02278-7 (PMC12856015; doi:10.1038/s41746-025-02278-7)
Supplement: Supplementary file 1 — Supplementary Information [file 41746_2025_2278_MOESM1_ESM.pdf]

Supplementary Table 1: consultation characteristics

	AHON	RUMC	MUMC+
Total	446,354	109,856	367,960
Male	188,770	46,518	155,040
Female	257,584	60,766	212,920
0	21,216	9475	14,542
1-3	41,923	15,051	32,913
4-5	16,078	5618	13,558
6-12	24,287	7689	19,477
13-17	20,299	4294	12,510
18-25	40,296	8194	24,874
26-40	59,606	17,746	50,874
41-55	63,845	16,483	56,732
56-70	78,059	14,782	75,273
71+	80,745	7952	67,207

Supplementary Table 1. Overview of consultation characteristics across datasets. The table presents the total number of consultations in the AHON, RUMC, and MUMC+ databases, stratified by sex (male, female) and age group (10 categories).

Supplementary Table 2: Full set of cluster visualizations generated by the ERNIE framework

Clusters from 1-2020 - 3-2020 (AHON)

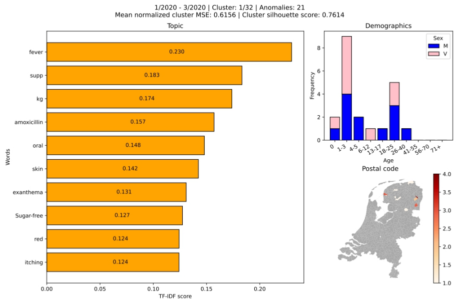

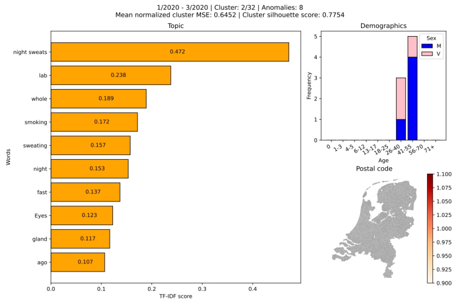

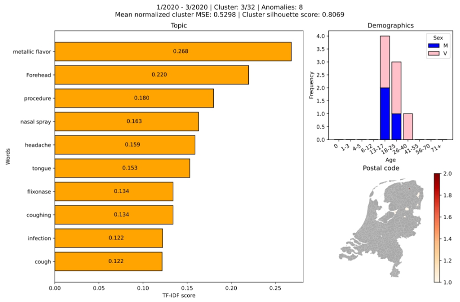

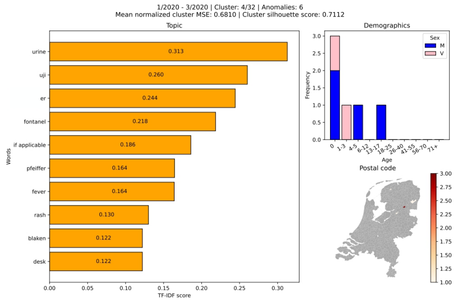

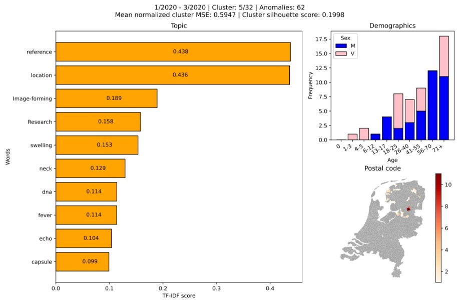

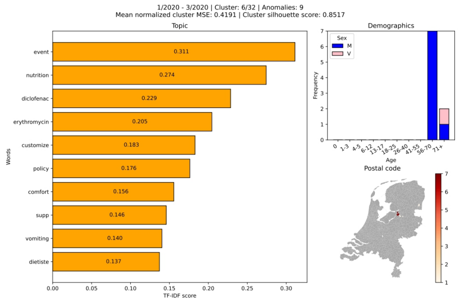

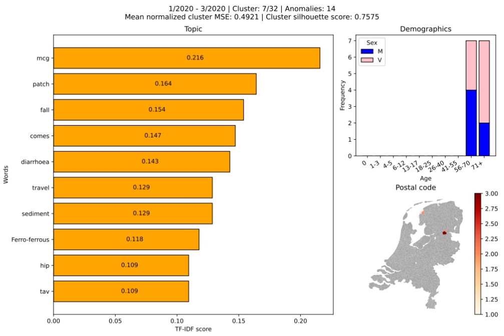
1/2020 - 3/2020 | Cluster: 7/32 | Anomalies: 14
Mean normalized cluster MSE: 0.4921 | Cluster silhouette score: 0.7575
Topic
mcg 0.216
patch 0.164
fall 0.154
comes 0.147
diarrhoea 0.143
travel 0.129
sediment 0.129
Ferro-ferrous 0.118
hip 0.109
tav 0.109
Words
TF-IDF score
Demographics
Sex
M
V
Frequency
Age
Postal code

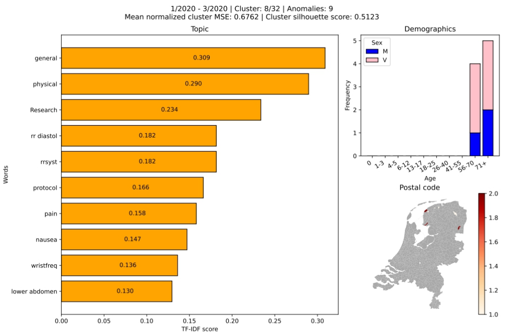
1/2020 - 3/2020 | Cluster: 8/32 | Anomalies: 9
Mean normalized cluster MSE: 0.6762 | Cluster silhouette score: 0.5123
Topic
general 0.309
physical 0.290
Research 0.234
rr diastol 0.182
rrsyst 0.182
protocol 0.166
pain 0.158
nausea 0.147
wristfreq 0.136
lower abdomen 0.130
Words
TF-IDF score
Demographics
Sex
M
V
Frequency
Age
Postal code

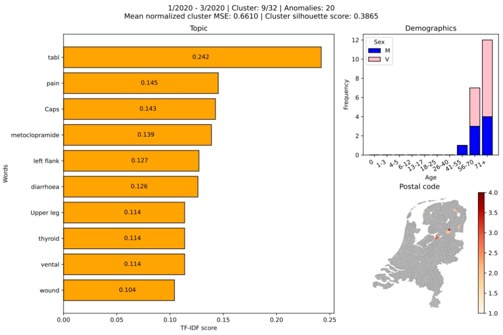
1/2020 - 3/2020 | Cluster: 9/32 | Anomalies: 20
Mean normalized cluster MSE: 0.6610 | Cluster silhouette score: 0.3865
Topic
tabl 0.242
pain 0.145
Caps 0.143
metoclopramide 0.139
left flank 0.127
diarrhoea 0.126
Upper leg 0.114
thyroid 0.114
vental 0.114
wound 0.104
Words
TF-IDF score
Demographics
Sex
M
V
Frequency
Age
Postal code

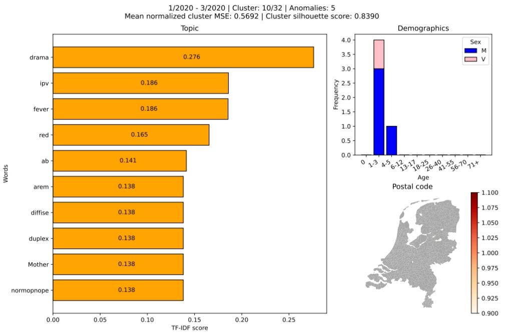
1/2020 - 3/2020 | Cluster: 10/32 | Anomalies: 5
Mean normalized cluster MSE: 0.5692 | Cluster silhouette score: 0.8390
Topic
drama 0.276
ipv 0.186
fever 0.186
red 0.165
ab 0.141
arem 0.138
diffise 0.138
duplex 0.138
Mother 0.138
normopnope 0.138
Words
TF-IDF score
Demographics
Sex
M
V
Frequency
Age
Postal code

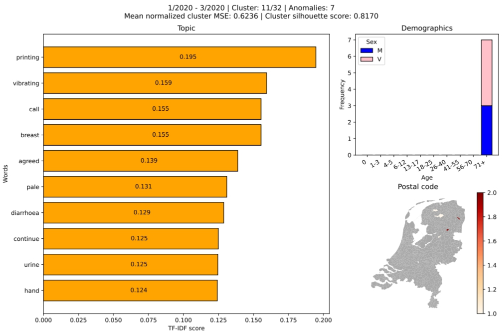
1/2020 - 3/2020 | Cluster: 11/32 | Anomalies: 7
Mean normalized cluster MSE: 0.6236 | Cluster silhouette score: 0.8170
Topic
printing 0.195
vibrating 0.159
call 0.155
breast 0.155
agreed 0.139
pale 0.131
diarrhoea 0.129
continue 0.125
urine 0.125
hand 0.124
Words
TF-IDF score
Demographics
Sex
M
V
Frequency
Age
Postal code

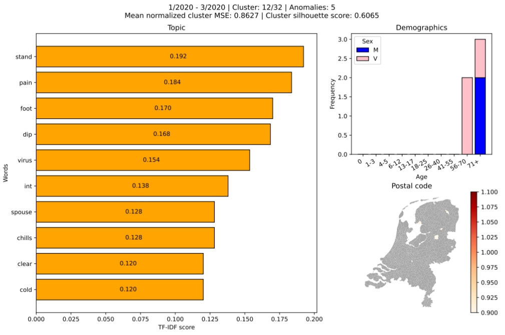
1/2020 - 3/2020 | Cluster: 12/32 | Anomalies: 5
Mean normalized cluster MSE: 0.8627 | Cluster silhouette score: 0.6065
Topic
stand 0.192
pain 0.184
foot 0.170
dip 0.168
virus 0.154
int 0.138
spouse 0.128
chills 0.128
clear 0.120
cold 0.120
Words
TF-IDF score
Demographics
Sex
M
V
Frequency
Age
Postal code

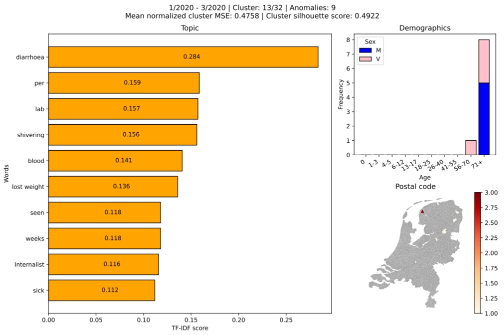
1/2020 - 3/2020 | Cluster: 13/32 | Anomalies: 9
Mean normalized cluster MSE: 0.4758 | Cluster silhouette score: 0.4922
Topic
diarrhoea 0.284
per 0.159
lab 0.157
shivering 0.156
blood 0.141
lost weight 0.136
seen 0.118
weeks 0.118
Internalist 0.116
sick 0.112
Words
TF-IDF score
Demographics
Sex
M
V
Frequency
Age
Postal code

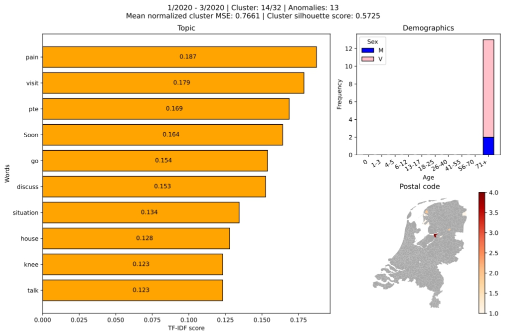
1/2020 - 3/2020 | Cluster: 14/32 | Anomalies: 13
Mean normalized cluster MSE: 0.7661 | Cluster silhouette score: 0.5725
Topic
pain 0.187
visit 0.179
pte 0.169
Soon 0.164
go 0.154
discuss 0.153
situation 0.134
house 0.128
knee 0.123
talk 0.123
Words
TF-IDF score
Demographics
Sex
M
V
Frequency
Age
Postal code

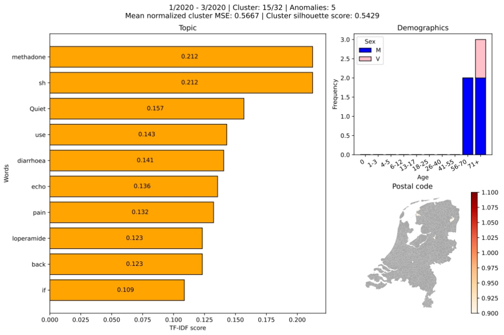
1/2020 - 3/2020 | Cluster: 15/32 | Anomalies: 5
Mean normalized cluster MSE: 0.5667 | Cluster silhouette score: 0.5429
Topic
methadone 0.212
sh 0.212
Quiet 0.157
use 0.143
diarrhoea 0.141
echo 0.136
pain 0.132
loperamide 0.123
back 0.123
if 0.109
Words
TF-IDF score
Demographics
Sex
M
V
Frequency
Age
Postal code

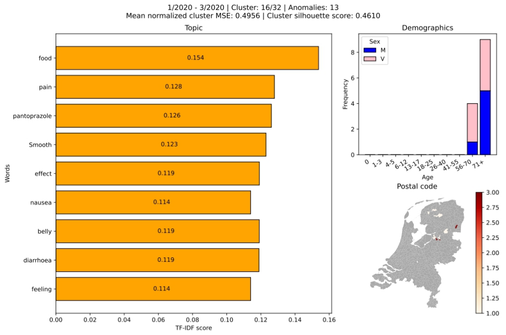
1/2020 - 3/2020 | Cluster: 16/32 | Anomalies: 13
Mean normalized cluster MSE: 0.4956 | Cluster silhouette score: 0.4610
Topic
food 0.154
pain 0.128
pantoprazole 0.126
Smooth 0.123
effect 0.119
nausea 0.114
belly 0.119
diarrhoea 0.119
feeling 0.114
Words
TF-IDF score
Demographics
Sex
M
V
Frequency
Age
Postal code

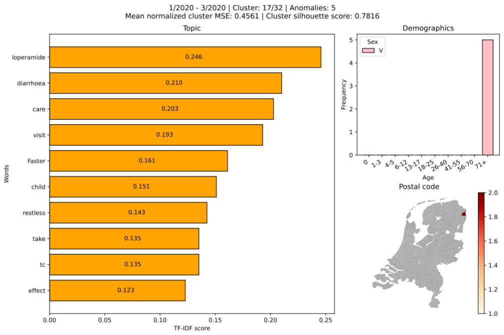
1/2020 - 3/2020 | Cluster: 17/32 | Anomalies: 5
Mean normalized cluster MSE: 0.4561 | Cluster silhouette score: 0.7816
Topic
loperamide 0.246
diarrhoea 0.210
care 0.203
visit 0.193
Faster 0.161
child 0.151
restless 0.143
take 0.135
tc 0.135
effect 0.123
Words
TF-IDF score
Demographics
Sex
V
Frequency
Age
Postal code

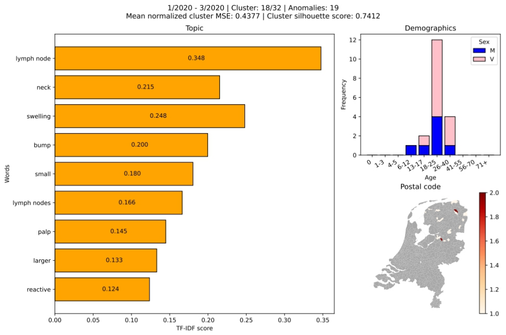
1/2020 - 3/2020 | Cluster: 18/32 | Anomalies: 19
Mean normalized cluster MSE: 0.4377 | Cluster silhouette score: 0.7412
Topic
lymph node 0.348
neck 0.215
swelling 0.248
bump 0.200
small 0.180
lymph nodes 0.166
palp 0.145
larger 0.133
reactive 0.124
Words
TF-IDF score
Demographics
Sex
M
V
Frequency
Age
Postal code

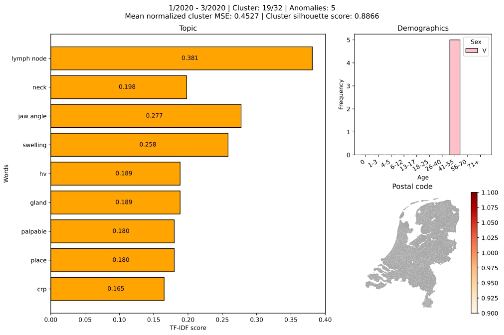
1/2020 - 3/2020 | Cluster: 19/32 | Anomalies: 5
Mean normalized cluster MSE: 0.4527 | Cluster silhouette score: 0.8866
Topic
lymph node 0.381
neck 0.198
jaw angle 0.277
swelling 0.258
hv 0.189
gland 0.189
palpable 0.180
place 0.180
crp 0.165
Words
TF-IDF score
Demographics
Sex
V
Frequency
Age
Postal code

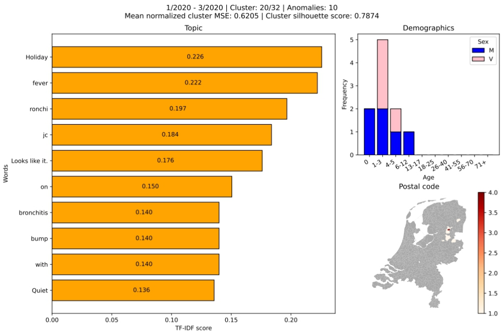
1/2020 - 3/2020 | Cluster: 20/32 | Anomalies: 10
Mean normalized cluster MSE: 0.6205 | Cluster silhouette score: 0.7874
Topic
Holiday 0.226
fever 0.222
ronchi 0.197
jc 0.184
Looks like it. 0.176
on 0.150
bronchitis 0.140
bump 0.140
with 0.140
Quiet 0.136
Words
TF-IDF score
Demographics
Sex
M
V
Frequency
Age
Postal code

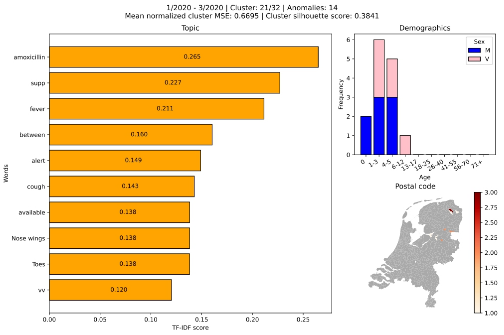
1/2020 - 3/2020 | Cluster: 21/32 | Anomalies: 14
Mean normalized cluster MSE: 0.6695 | Cluster silhouette score: 0.3841
Topic
amoxicillin 0.265
supp 0.227
fever 0.211
between 0.160
alert 0.149
cough 0.143
available 0.138
Nose wings 0.138
Toes 0.138
vv 0.120
Words
TF-IDF score
Demographics
Sex
M
V
Frequency
Age
Postal code

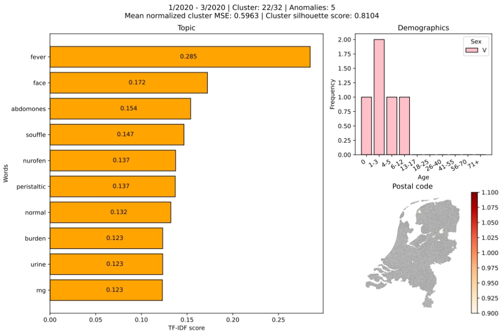
1/2020 - 3/2020 | Cluster: 22/32 | Anomalies: 5
Mean normalized cluster MSE: 0.5963 | Cluster silhouette score: 0.8104
Topic
fever 0.285
face 0.172
abdomones 0.154
souffle 0.147
nurofen 0.137
peristaltic 0.137
normal 0.132
burden 0.123
urine 0.123
mg 0.123
Words
TF-IDF score
Demographics
Sex
V
Frequency
Age
Postal code

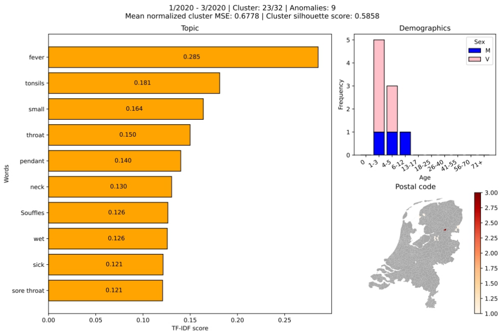
1/2020 - 3/2020 | Cluster: 23/32 | Anomalies: 9
Mean normalized cluster MSE: 0.6778 | Cluster silhouette score: 0.5858
Topic
fever 0.285
tonsils 0.181
small 0.164
throat 0.150
pendant 0.140
neck 0.130
Souffles 0.126
wet 0.126
sick 0.121
sore throat 0.121
Words
TF-IDF score
Demographics
Sex
M
V
Frequency
Age
Postal code

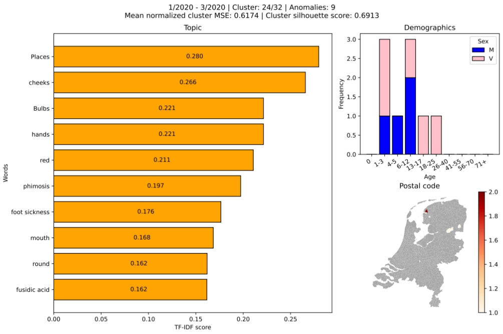
1/2020 - 3/2020 | Cluster: 24/32 | Anomalies: 9
Mean normalized cluster MSE: 0.6174 | Cluster silhouette score: 0.6913
Topic
Places 0.280
cheeks 0.266
Bulbs 0.221
hands 0.221
red 0.211
phimosis 0.197
foot sickness 0.176
mouth 0.168
round 0.162
fusidic acid 0.162
Words
TF-IDF score
Demographics
Sex
M
V
Frequency
Age
Postal code

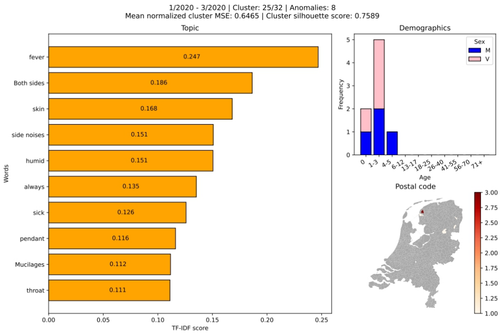
1/2020 - 3/2020 | Cluster: 25/32 | Anomalies: 8
Mean normalized cluster MSE: 0.6465 | Cluster silhouette score: 0.7589
Topic
Demographics
Postal code
fever 0.247
Both sides 0.186
skin 0.168
side noises 0.151
humid 0.151
always 0.135
sick 0.126
pendant 0.116
Mucilages 0.112
throat 0.111
Words
TF-IDF score
Frequency
Age
Sex
M
V

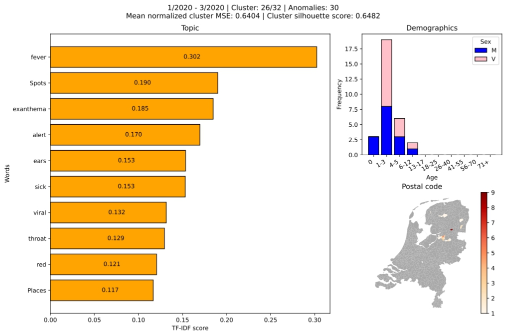
1/2020 - 3/2020 | Cluster: 26/32 | Anomalies: 30
Mean normalized cluster MSE: 0.6404 | Cluster silhouette score: 0.6482
Topic
Demographics
Postal code
fever 0.302
Spots 0.190
exanthema 0.185
alert 0.170
ears 0.153
sick 0.153
viral 0.132
throat 0.129
red 0.121
Places 0.117
Words
TF-IDF score
Frequency
Age
Sex
M
V

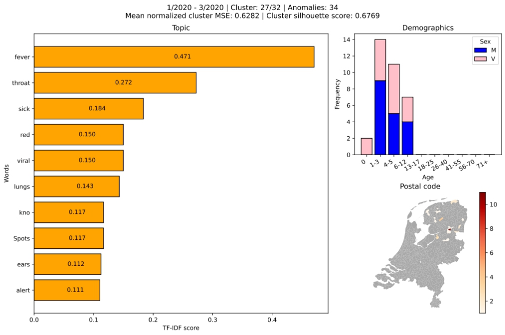
1/2020 - 3/2020 | Cluster: 27/32 | Anomalies: 34
Mean normalized cluster MSE: 0.6282 | Cluster silhouette score: 0.6769
Topic
Demographics
Postal code
fever 0.471
throat 0.272
sick 0.184
red 0.150
viral 0.150
lungs 0.143
kno 0.117
Spots 0.117
ears 0.112
alert 0.111
Words
TF-IDF score
Frequency
Age
Sex
M
V

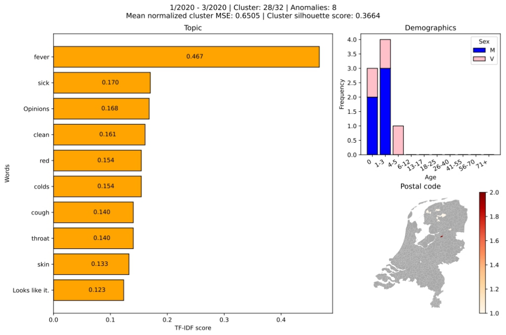
1/2020 - 3/2020 | Cluster: 28/32 | Anomalies: 8
Mean normalized cluster MSE: 0.6505 | Cluster silhouette score: 0.3664
Topic
Demographics
Postal code
fever 0.467
sick 0.170
Opinions 0.168
clean 0.161
red 0.154
colds 0.154
cough 0.140
throat 0.140
skin 0.133
Looks like it. 0.123
Words
TF-IDF score
Frequency
Age
Sex
M
V

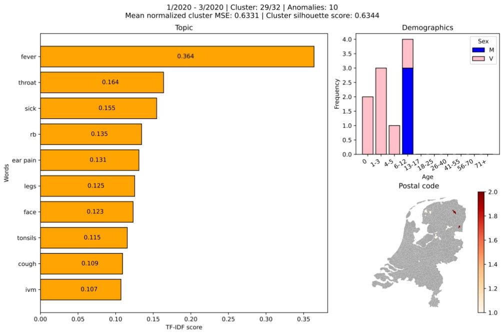
1/2020 - 3/2020 | Cluster: 29/32 | Anomalies: 10
Mean normalized cluster MSE: 0.6331 | Cluster silhouette score: 0.6344
Topic
Demographics
Postal code
fever 0.364
throat 0.164
sick 0.155
rb 0.135
ear pain 0.131
legs 0.125
face 0.123
tonsils 0.115
cough 0.109
ivm 0.107
Words
TF-IDF score
Frequency
Age
Sex
M
V

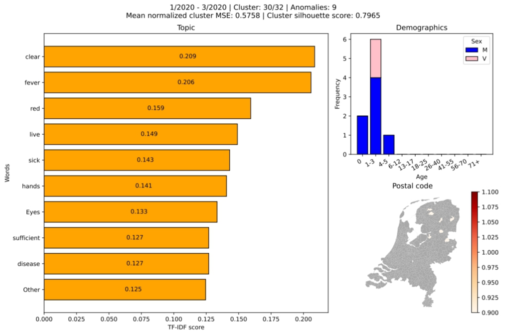
1/2020 - 3/2020 | Cluster: 30/32 | Anomalies: 9
Mean normalized cluster MSE: 0.5758 | Cluster silhouette score: 0.7965
Topic
Demographics
Postal code
clear 0.209
fever 0.206
red 0.159
live 0.149
sick 0.143
hands 0.141
Eyes 0.133
sufficient 0.127
disease 0.127
Other 0.125
Words
TF-IDF score
Frequency
Age
Sex
M
V

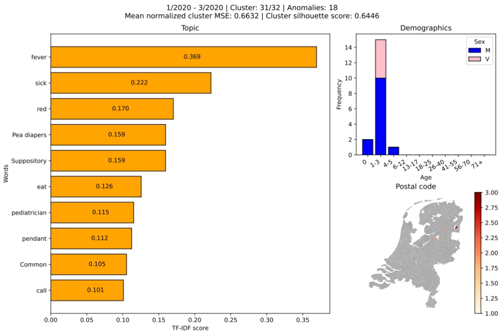
1/2020 - 3/2020 | Cluster: 31/32 | Anomalies: 18
Mean normalized cluster MSE: 0.6632 | Cluster silhouette score: 0.6446
Topic
Words
fever
0.369
sick
0.222
red
0.170
Pea diapers
0.159
Suppository
0.159
eat
0.126
pediatrician
0.115
pendant
0.112
Common
0.105
call
0.101
TF-IDF score
Demographics
Sex
M
V
Frequency
Age
0
1-3
4-5
6-12
13-17
18-25
26-40
41-55
56-70
71+
Postal code

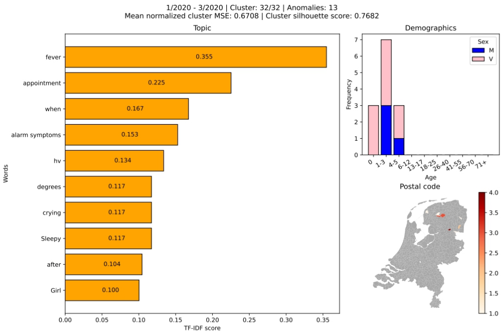
1/2020 - 3/2020 | Cluster: 32/32 | Anomalies: 13
Mean normalized cluster MSE: 0.6708 | Cluster silhouette score: 0.7682
Topic
Words
fever
0.355
appointment
0.225
when
0.167
alarm symptoms
0.153
hv
0.134
degrees
0.117
crying
0.117
Sleepy
0.117
after
0.104
Girl
0.100
TF-IDF score
Demographics
Sex
M
V
Frequency
Age
0
1-3
4-5
6-12
13-17
18-25
26-40
41-55
56-70
71+
Postal code

Clusters from 2-2020 - 3-2020 (AHON)

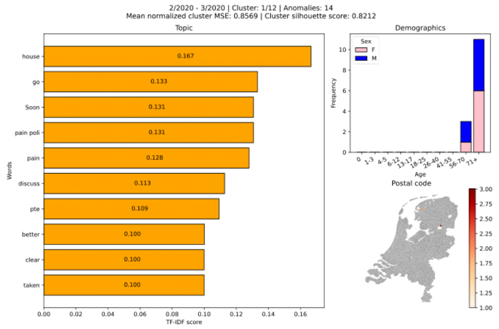

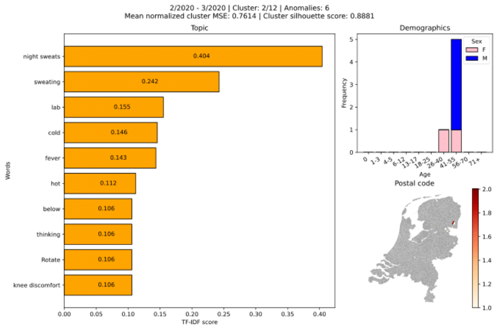

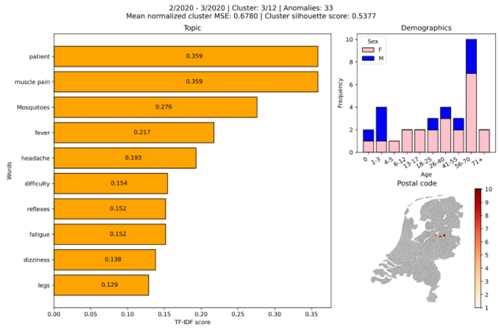

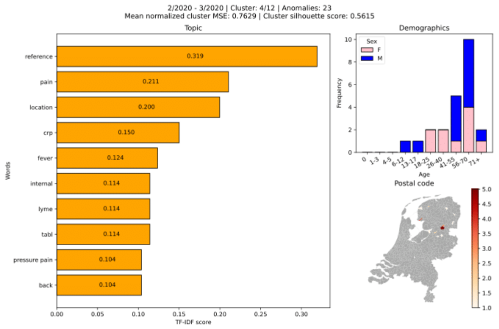

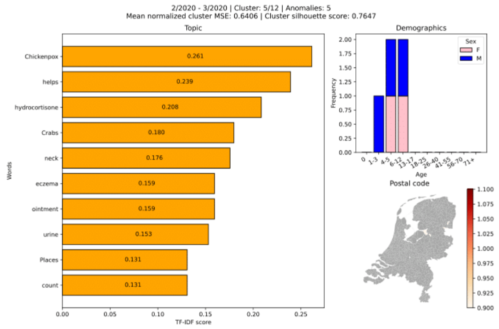

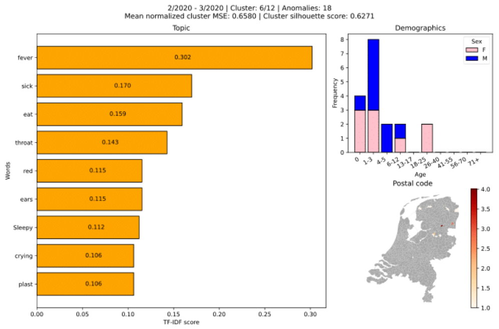

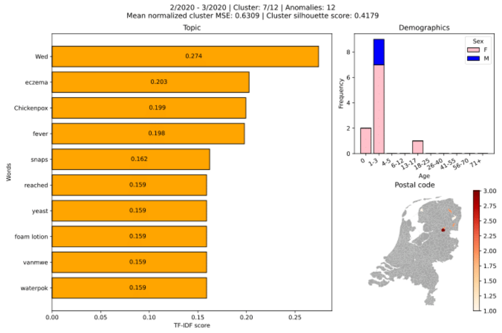
2/2020 - 3/2020 | Cluster: 7/12 | Anomalies: 12
Mean normalized cluster MSE: 0.6309 | Cluster silhouette score: 0.4179
Topic
Wed 0.274
eczema 0.203
Chickenpox 0.199
fever 0.198
snaps 0.162
reached 0.159
yeast 0.159
foam lotion 0.159
vanmwe 0.159
waterpok 0.159
Words
TF-IDF score
Demographics
Sex
F
M
Frequency
Age
Postal code

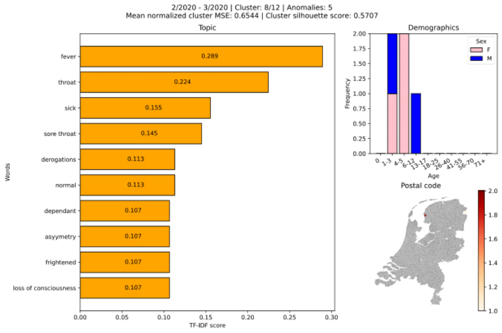
2/2020 - 3/2020 | Cluster: 8/12 | Anomalies: 5
Mean normalized cluster MSE: 0.6544 | Cluster silhouette score: 0.5707
Topic
fever 0.289
throat 0.224
sick 0.155
sore throat 0.145
derogations 0.113
normal 0.113
dependant 0.107
asyymetry 0.107
frightened 0.107
loss of consciousness 0.107
Words
TF-IDF score
Demographics
Sex
F
M
Frequency
Age
Postal code

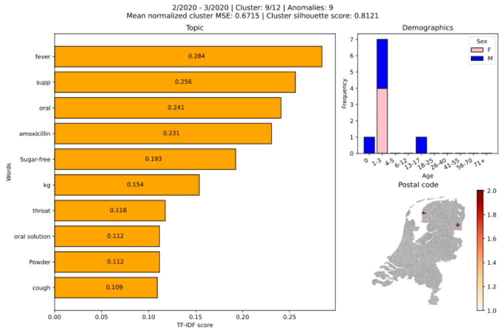
2/2020 - 3/2020 | Cluster: 9/12 | Anomalies: 9
Mean normalized cluster MSE: 0.6715 | Cluster silhouette score: 0.8121
Topic
fever 0.284
supp 0.256
oral 0.241
amoxicillin 0.231
Sugar-free 0.193
kg 0.154
throat 0.118
oral solution 0.112
Powder 0.112
cough 0.109
Words
TF-IDF score
Demographics
Sex
F
M
Frequency
Age
Postal code

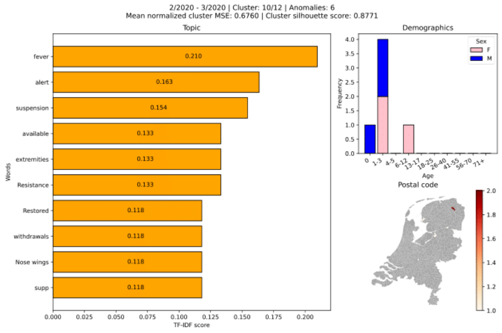
2/2020 - 3/2020 | Cluster: 10/12 | Anomalies: 6
Mean normalized cluster MSE: 0.6760 | Cluster silhouette score: 0.8771
Topic
fever 0.210
alert 0.163
suspension 0.154
available 0.133
extremities 0.133
Resistance 0.133
Restored 0.118
withdrawals 0.118
Nose wings 0.118
supp 0.118
Words
TF-IDF score
Demographics
Sex
F
M
Frequency
Age
Postal code

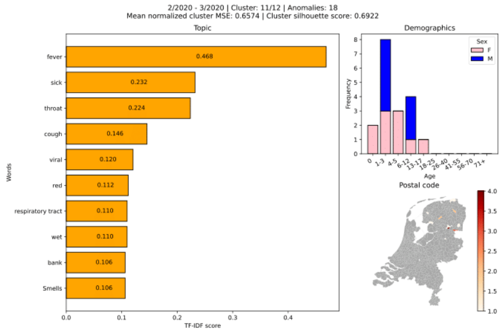
2/2020 - 3/2020 | Cluster: 11/12 | Anomalies: 18
Mean normalized cluster MSE: 0.6574 | Cluster silhouette score: 0.6922
Topic
fever 0.468
sick 0.232
throat 0.224
cough 0.146
viral 0.120
red 0.112
respiratory tract 0.110
wet 0.110
bank 0.106
Smells 0.106
Words
TF-IDF score
Demographics
Sex
F
M
Frequency
Age
Postal code

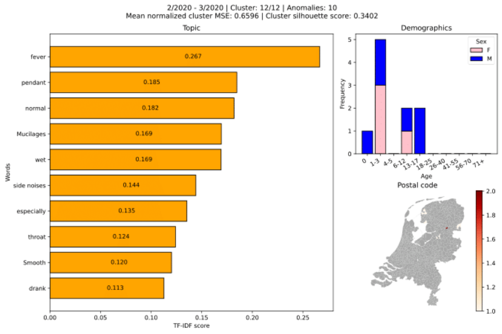
2/2020 - 3/2020 | Cluster: 12/12 | Anomalies: 10
Mean normalized cluster MSE: 0.6596 | Cluster silhouette score: 0.3402
Topic
fever 0.267
pendant 0.185
normal 0.182
Mucilages 0.169
wet 0.169
side noises 0.144
especially 0.135
throat 0.124
Smooth 0.120
drank 0.113
Words
TF-IDF score
Demographics
Sex
F
M
Frequency
Age
Postal code

Clusters from 3-2020 - 4-2020 (AHON)

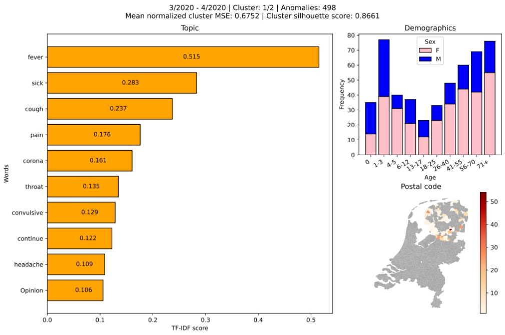

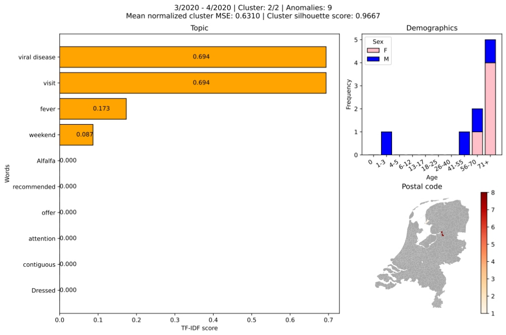

Clusters from 7-2021 - 8-2021 (AHON)

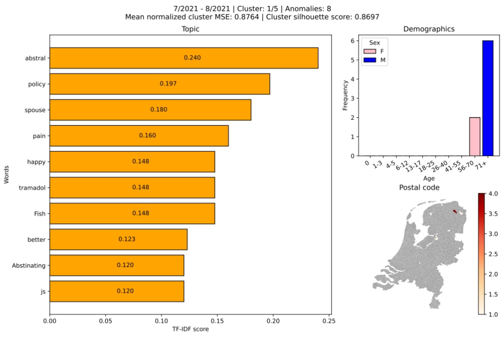

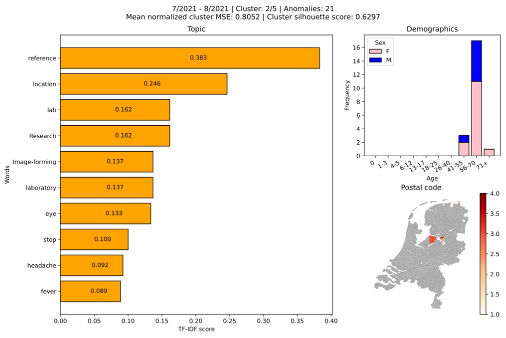

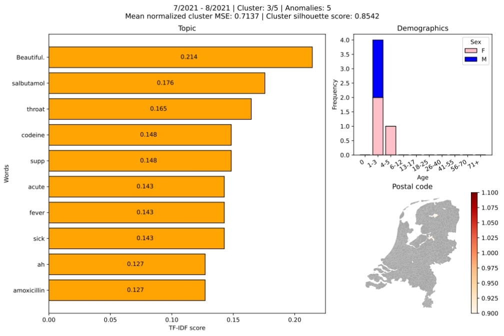

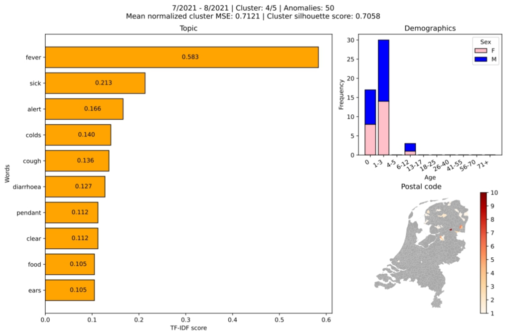

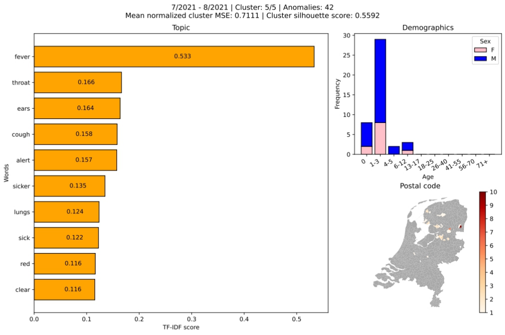

Clusters from 7-2021 - 8-2021 (MUMC)

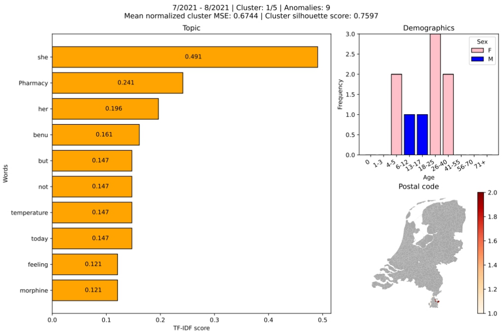

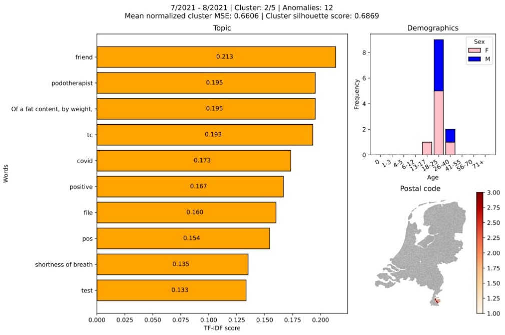

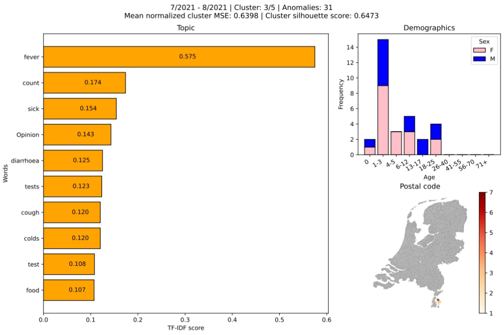

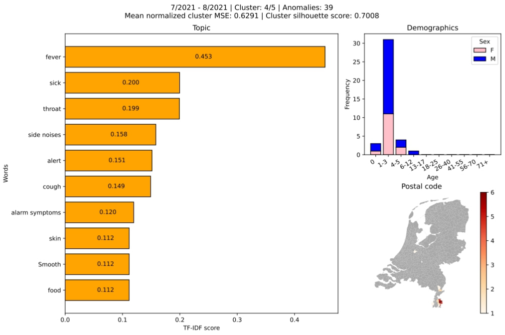

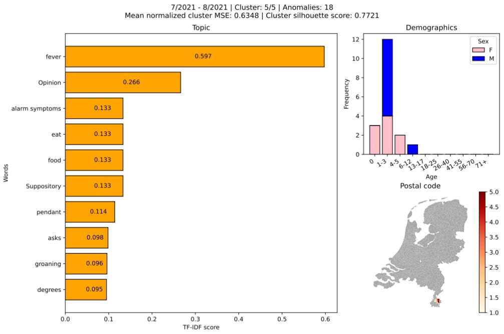

Clusters from 7-2021 - 8-2021 (RUMC)

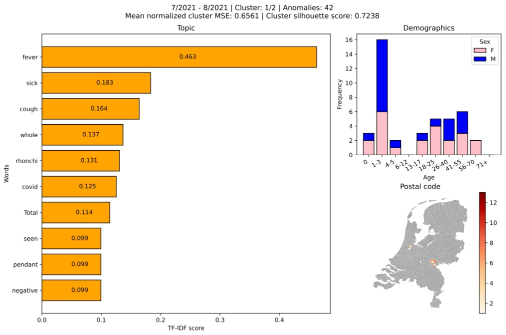

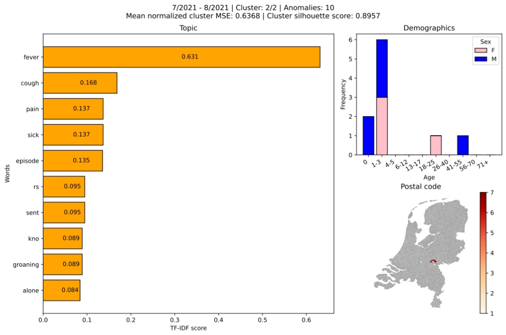

Supplementary Table 3: ICPC list

ICPC 1	Explanation 1	ICPC 2	Explanation 2	ICPC 3	Explanation 3
A01	Pain all over the body	D71	Mumps	R09	Sinus complaints
A02	Chills	D72.01	Liver inflammation due to virus (hepatitis A)	R21	Throat complaints
A03	Fever	D73	Gastrointestinal infection	R21.01	Sore throat
A09.01	Night sweats	D87	Stomach problem	R22	Tonsil complaints
A71	Measles	D87.01	Stomach/duodenal inflammation	R23	Voice complaints
A72	Chickenpox	D87.02	Stomach complaints	R24	Coughing up blood
A73	Malaria	H04	Ear discharge	R28	Airway problems
A74	Rubella	H71	Middle ear/eardrum inflammation	R29	Respiratory complaints
A75	Infectious mononucleosis	H72	Fluid behind eardrum (glue ear)	R70	Tuberculosis (TB)
A76	Viral disease with rash	N01	Headache	R71	Whooping cough
A76.01	Sixth disease	N07	(Fever) seizures	R72	Bacterial throat infection/scarlet fever
A76.02	Fifth disease	N07.01	Febrile seizures	R72.01	Bacterial throat infection
A76.03	Hand-foot-mouth disease	N16	Problems with smell/taste	R72.02	Scarlet fever
A77	Viral diseases	N17	Dizziness	R74	Acute upper respiratory infection (nose/mouth/throat/sinuses)
A78	Infectious diseases	N17.01	Vertigo (feeling like everything is spinning or moving)	R74.01	Common cold
A78.05	Lyme disease (tick-borne disease)	N17.02	Light-headedness	R74.02	Acute pharyngitis
A92	Inflammation due to a parasite (toxoplasmosis)	N70	Polio/infection from another virus	R75	Acute/chronic sinusitis
B02	Enlarged lymph nodes	N70.01	Acute polio	R75.01	Acute sinusitis
B03	Lymph node complaints	N71	Brain (meningeal) inflammation	R75.02	Chronic sinusitis
B70	Acute lymph node inflammation	N71.01	Bacterial meningitis	R76	Acute tonsillitis
B86.01	Elevated inflammation value in blood (ESR)	N71.02	Viral meningitis	R76.01	Acute tonsillitis
D09	Nausea	N71.03	Brain inflammation	R76.02	Pus-filled abscess in the throat
D10	Vomiting	N71.04	Spinal cord inflammation	R77	Acute laryngitis/vocal cord/tracheal inflammation
D11	diarrhoea	N72	Tetanus	R77.01	Viral airway infection with breathing problems and barking cough (croup)
D22	Worms/other parasites	N73	Infection of brain/spinal cord/nerves	R77.02	Acute epiglottitis
D22.01	Pinworms	N91	Facial muscle paralysis (Bell's palsy)	R78	Acute airway inflammation
D22.02	Roundworm	N94.01	Guillain-Barré syndrome	R80	Influenza
D22.03	Tapeworm	R01	Pain from airways	R81	Pneumonia
D29	Gastrointestinal complaints	R02	Shortness of breath	R81.01	Legionella pneumonia
D70	Diarrhoea due to bacteria or parasites	R03	Wheezing	R82	Pleurisy
D70.01	Intestinal infection due to Salmonella bacteria	R04	Breathing problems	R83	Respiratory infection
D70.02	Intestinal infection due to bacteria	R05	Coughing	R83.01	Diphtheria
D70.03	Intestinal inflammation due to Giardia parasite	R07	Sneezing/nasal congestion/runny nose	R83.03	SARS-CoV-2 (COVID-19)
D70.04	Intestinal inflammation due to Amoeba parasite	R08	Nasal complaints	T70	Gland infection

Supplementary Table 4: technical specifications of all models

Anomaly Detection Model

Input Features

- Text embeddings: Dimensionality = 768.
- ICPC-codes: One-hot encoded and embedded to 20 dimensions.
- Demographic features: Standardised age and one-hot encoded gender.
- Temporal features: Sinusoidal-encoded month.

Architecture

Attention Layers:

- Layer 1: Refines relationships between text embeddings and ICPC-codes.
- Layer 2: Integrates output from Layer 1 with demographic and temporal features.

Encoder:

- Fully connected layers: 256 → 128 → 64 units with ReLU activations.
- Latent space: 32 units for compact representation.

Decoder:

- Fully connected layers: 64 → 128 → 256 units to reconstruct input.
- Output layer: Linear activation, dimensions matching the input.

Training

- Loss function: Mean Squared Error (MSE).
- Optimizer: Adam with a learning rate of 0.001.
- Batch size: 32.
- Epochs: 5 with validation split (20%).

Anomaly Scoring

- Reconstruction error (MSE) is used to compute an anomaly score.
- Threshold: 90th percentile of the training reconstruction errors.

Dimensionality Reduction:

UMAP:

- Parameters: n_neighbors=15, min_dist=0.1, metric='cosine'.
- Output dimensions: 50.

Clustering:

HDBSCAN:

- Parameters: min_cluster_size=5, min_samples=8, metric='precomputed', cluster_selection_method='eom'.

- Distance matrix: Cosine similarity.

Cluster Analysis:

Clusters are characterised using:

- TF-IDF scores of keywords.
- Demographic distributions (age, gender, postal code).
- Mean silhouette score to evaluate cluster quality.
- Mean normalised MSE-score to evaluate anomalousness.
